# Heterologous Expression and Characterization of A Novel Ochratoxin A Degrading Enzyme, N-acyl-L-amino Acid Amidohydrolase, from *Alcaligenes faecalis*

**DOI:** 10.3390/toxins11090518

**Published:** 2019-09-06

**Authors:** Honghai Zhang, Yunpeng Zhang, Tie Yin, Jing Wang, Xiaolin Zhang

**Affiliations:** 1 Gansu Key Laboratory of Viticulture and Enology, College of Food Science and Engineering, Gansu Agricultural University, Lanzhou 730070, China; 2 Academy of State, Administration of Grain, Beijing 100032, China (Y.Z.) (T.Y.); 3 Beijing Key Laboratory of Nutrition and Health and Food Safety, COFCO Nutrition and Health Institute, Beijing 102209, China

**Keywords:** ochratoxin A, degradation, amidohydrolase, *Alcaligenes faecalis*, mycotoxin

## Abstract

Ochratoxin A (OTA) is a well-known, natural contaminant in foods and feeds because of its toxic effects, such as nephrotoxicity in various animals. Recent studies have revealed that *Alcaligenes faecalis* could generate enzymes to efficiently degrade OTA to ochratoxin α (OTα) in vitro. In an effort to obtain the OTA degrading mechanism, we purified and identified a novel degrading enzyme, N-acyl-L-amino acid amidohydrolase (*Af*OTase), from *A. faecalis* DSM 16503 via mass spectrometry. The same gene of the enzyme was also encountered in other *A. faecalis* strains. *Af*OTase belongs to peptidase family M20 and contains metal ions at the active site. In this study, recombination *Af*OTase was expressed and characterized in *Escherichia coli*. The molecular mass of recombinant r*Af*OTase was approximately 47.0 kDa, as determined by sodium dodecyl sulfate-polyacrylamide gel electrophoresis (SDS-PAGE). The enzyme exhibited a wide temperature range (30–70 °C) and pH adaptation (4.5–9.0) and the optimal temperature and pH were 50 °C and 6.5, respectively.

## 1. Introduction

Ochratoxin A (OTA) is a natural, toxic, secondary metabolite produced by several fungal species of the genera *Penicillium* and *Aspergillus* [1]. OTA spreads widely in, for example, cereals, corn, grape, wine, beer, fish, pork, seasoning, oilseeds, and edible oils. In areas with a hot and humid climate, OTA contamination has a higher incidence because of the suitable conditions for the growth of toxigenic fungi. Owing to the physical and chemical stability of OTA, it is hard to be removed or degraded in agricultural commodities [2]. Thus, there may be contamination risk in each step of the agricultural supply chain, including pre-harvest, post-harvest, and during processing and storage [3,4,5].

OTA, the effects of which are mainly observed in the kidney and liver, is one of the most dangerous mycotoxins for humans and animals [6]. Nephrotoxicity, hepatotoxicity, carcinogenicity, and teratogenicity are the most relevant toxic effects [7,8]. Many researchers have suggested that OTA exposure is responsible for several human diseases, including Balkan endemic nephropathy [9]. It has also been considered that OTA has genotoxicity and immunotoxicity [10,11].

As a foodborne hazard, the presence of OTA in agricultural and sideline products has attracted great public attention and the food safety regulatory departments have recently enforced the controls of its legal limitation [12]. Therefore, to reduce the risk of human and animal exposure, several physical, chemical, and microbiological strategies were proposed to eliminate the hazard [13]. Among these strategies, biological methods using microorganisms or enzymes are more efficient, because they have high efficiency and high target specificity and are environmentally friendly, and they can preserve the nutritious value of food and feed products [14].

**Figure 1:**
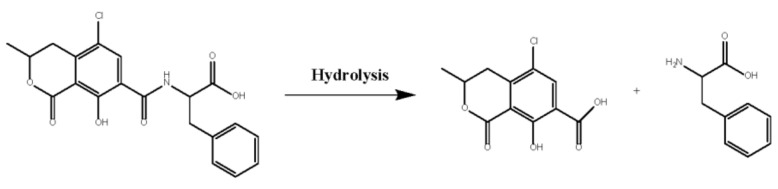
Enzymatic degradation of ochratoxin A via hydrolysis of its amide bond using N-acyl-L-amino acid amidohydrolase from *Alcaligenes faecalis*.

Adsorption and biodegradation are the main approaches for OTA detoxification by micro-organisms. *Lactobacillus* species and yeasts are the most studied microorganisms for OTA bio-sorption, which depends on the cell wall components and structure [15,16]. The biodegradation of OTA is implemented by hydrolysis, hydroxylation, lactone ring opening, and conjugation to other entities [17]. Among the final degradation products of this reaction, ochratoxin α (OTα), a product of the OTA amide bond hydrolysis reaction, is considered to be barely toxic or non-toxic (Figure 1). It is approximately 1000 times less toxic than OTA in brain cell cultures and its half-life in the blood is 10 times shorter than that of OTA (103 h) [18,19].

Recent studies have discovered that the *A. faecalis* strain is able to efficiently degrade OTA to OTα in vitro [20]. In an effort to obtain the OTA degrading mechanism, we purified a novel degrading enzyme from *A. faecalis* DSM 16503 and identified it as N-acyl-L-amino acid amidohydrolase (*Af*OTase) via mass spectrometry (unpublished data). The same gene of the enzyme is also encountered in other *A. faecalis* strains. In this study, the *Af*OTase gene was cloned, expressed, and purified in *Escherichia coli* BL21 (DE3), and the effects of temperature and pH on the catalytic activity of recombinant *Af*OTase (r*Af*OTase) were characterized. The hydrolysis products of ochratoxin A by r*Af*OTase are β-phenylalanine and ochratoxin A (Supplementary Figure S1).

## 2. Results and Discussion

### 2.1. Cloning and Amino Acid Sequencing of The AfOTase Gene from A. Faecalis DSM 16503

The sequence of the *Af*OTase gene from the gDNA of *A. faecalis* DSM 16305 contains a complete open reading frame (ORF) (1317 bp) that employs ATG and TAG as start and stop codons, respectively. The overall G+C content of the gene is 57%. The full-length gene encodes a polypeptide of 438 amino acid residues. The primers OD-F and OD-R allowed amplification of a 1251 bp long gene without the signal peptide, which consists of 22 amino acids, predicted by SignalP 4.0. The sequence of the cloned gene was deposited in GenBank accession no. OSZ37025.1.

**Figure 2:**
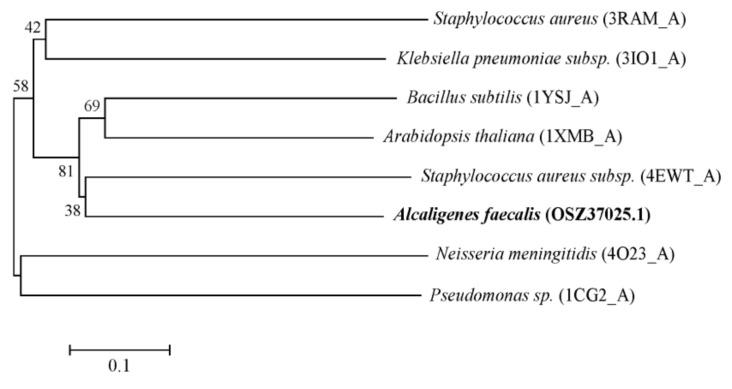
A phylogenetic tree was constructed based on the amino acid sequences of *Af*OTase by means of neighbor-joining analysis. Bootstrap values (n = 1000 replicates) are reported as percentages.

An amino acid homology analysis by BLASTP showed the highest identity (37%) with LAA-amino acid hydrolase from *Arabidopsis thaliana*, followed by aminohydrolase from methicillin-resistant *Staphylococcus aureus* (36%), Yxep protein from *Bacillus subtilis* (34%), aminobenzoyl-glutamate utilization protein from *Klebsiella pneumoniae* (25%), and Hmra from *S. aureus* (25%) (Figure 2). The enzyme belongs to the peptidase family M20 (clan MH) [21]. The peptidases of this clan have two catalytic zinc ions at the active site, bound by His/Asp, Asp, Glu, Asp/Glu, and His. This family comprises a peptidase dimerization domain that consists of four beta-strands and two alpha-helices which make up the dimerization surface.

Moreover, *Af*OTase has low homology in the amino acid sequence with other known OTA degrading enzymes, such as OTase from *A. niger* (Supplementary Figure S2).

### 2.2. Expression and Purification of rAfOTase

*Af*OTase gene was successfully cloned into pET-28a (+) vector (Supplementary Figure S3), confirmed by DNA sequencing and double digestion of the recombinant vector with BamHI and HindIII. The constructed pET-28a-r*Af*OTase vector was transformed and efficiently expressed in *E. coli* BL21 (DE3) intracellularly after it was induced with 0.5 mM isopropyl-beta-D-thiogalactopyranoside (IPTG) at 20 °C for 16 h.

The recombinant *Af*OTase was purified by a nitrilotriacetic acid complex of nickel (II) (Ni^2+^-NTA) resin affinity chromatography. The molecular mass of r*Af*OTase was slightly above 45.0 kDa as determined by SDS-PAGE (Supplementary Figure S4), and very close to the predicted molecular weight of the protein (~47.0 kDa).

### 2.3. Enzymatic Properties of rAfOTase

**Figure 3:**
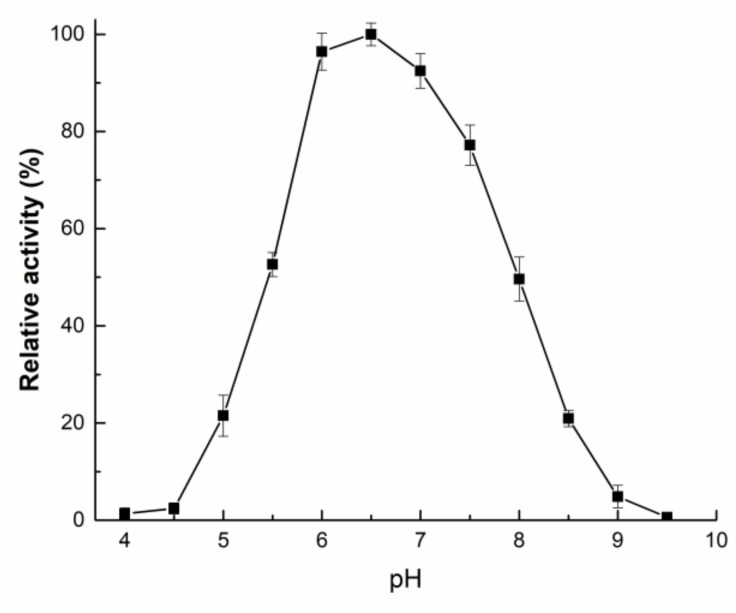
Effects of pH on r*Af*OTase catalytic activity. Reaction system consisted of 15 μg/mL r*Af*OTase, 20 mM buffer, and 1 μg/mL ochratoxin A.

**Figure 4:**
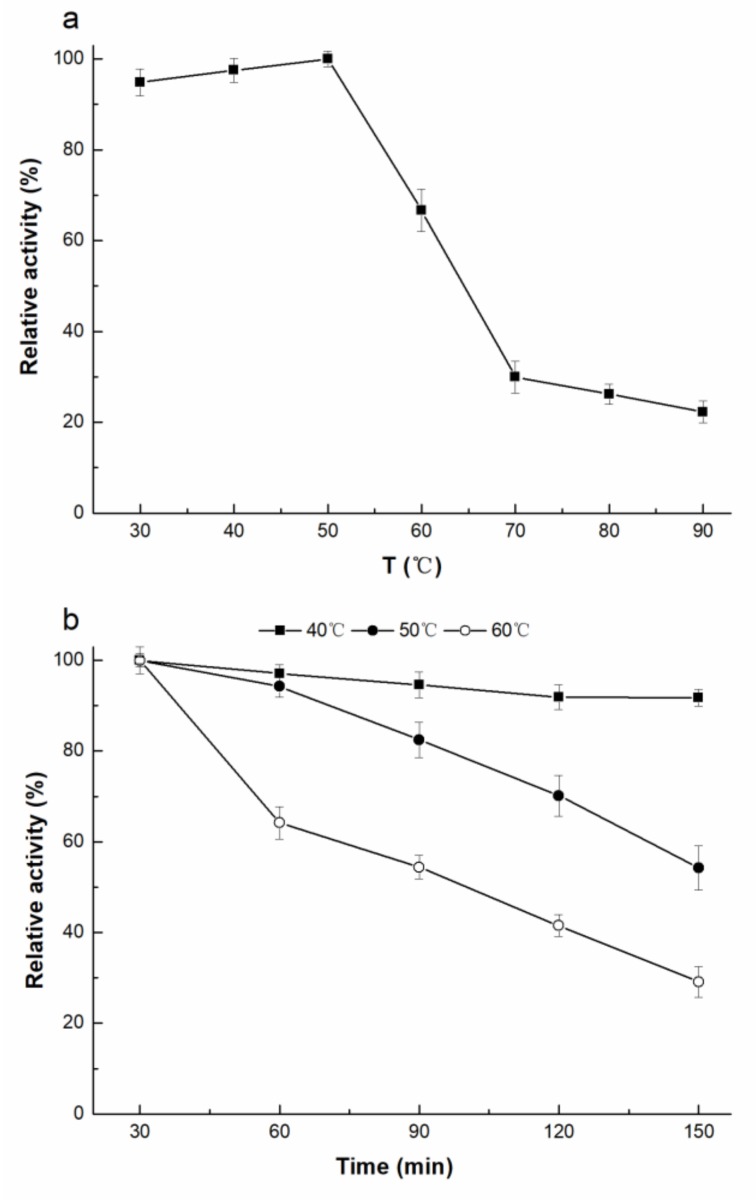
Effects of temperature on r*Af*OTase (**a**) catalytic activity and (**b**) stability. Reaction system consisted of 15 μg/mL r*Af*OTase, 20 mM buffer (pH 6.5), and 1 μg/mL ochratoxin A.

r*Af*OTase exhibits activity at various temperatures (30–70 °C) and pH values (4.5–9.0) (Figure 3). r*Af*OTase showed high levels of activity at pH 6.0 to 7.0 (> 90%), with an optimum pH of 6.5. When the pH was below 5.0 or above 8.5, the r*Af*OTase retained less than 20% of its optimal catalytic activity. The enzyme activity remained in varying degrees within 30–90 °C after incubation for 60 min with a substrate (Figure 4a). The optimal temperature for r*Af*OTase was 50 °C. r*Af*OTase was stable and retained no less than 90% of activity after it was incubated for 150 min at temperatures below 50 °C. After being incubated at 50 °C for 120 min, approximately 70% of activity was retained but the activity was substantially reduced at a temperature above 50 °C (Figure 4b).

Over the last two decades, only a few enzymes involved in the OTA degrading process have been purified and characterized [22,23]. Bovine pancreas carboxypeptidase A (CPA) (EC 3.4.17.1) was reported for the first time [24]. However, CPA has a weak degrading ability to detoxify completely. Subsequently, carboxypeptidase Y (CPY) (EC 3.4.16.1) from *Saccharomyces cerevisiae* was found but its degrading activity is very low, with optimal activity at pH 5.6 and 37 °C [13]. Further, similar enzymes are reported in different strains [23,25] and a few commercial proteases from *Aspergillus niger* are reported to hydrolyze OTA to OTα. For example, protease A and pancreatin could degrade 87.3% and 43.4%, respectively, of OTA (at 1 μg/mL) after 25 h at pH 7.5 and 37 °C [26].

Remarkably, Stander, et al. [27] first reported that Amano^TM^ lipase from *A. niger* had ochratoxin-degrading activity. Subsequent research showed that the degrading enzyme from *A. niger* is a metalloenzyme and more efficient than CPA [26,28]. Further purification and analysis of the product revealed that the active enzyme is an amidase, named ochratoxinase (OTase), and its hydrolytic activity is approximately 600 times higher than CPA in OTA degradation at pH 7.5 after being incubated for 60 min at 37 °C [29].

The *Af*OTase from *A. faecalis* exhibited not only efficient detoxification ability but also relatively wide pH and mesophilic stability when compared to other enzymes, as described above. However, for large-scale production of r*Af*OTase for industrial applications, a more cost-effective expression system needs to be employed and the pH and thermal stability should be enhanced.

## 3. Conclusions

In conclusion, *Af*OTase, a novel OTA degrading enzyme from *A. faecalis*, was cloned and produced using an *E. coli* expression system. It showed commendable thermal and pH stability. These important properties of *Af*OTase make it a novel potential candidate for commercial applications, such as detoxifying OTA in food or feed processing. Further study of this enzyme will be focused on the meaningful value of detoxifying OTA in the food and feed industries.

## 4. Materials and Methods

### 4.1. Strain and Growth Conditions

*Alcaligenes faecalis* DSM 16503, *Escherichia coli* Top10, and *E. coli* BL21 (DE3) were aerobically grown at 37 °C in Luria–Bertani (LB) medium. When necessary, 100 μg/mL ampicillin or 50 μg/mL kanamycin was added to the medium as screening pressure.

### 4.2. DNA Isolation and Sequence Data Analysis

Genomic DNA was extracted from *A. faecalis* DSM 16503 as described by Lee and Taylor [30]. Plasmid DNA from *E. coli* was purified by using the SanPrep Column Plasmid Mini-Preps Kit (Sangon Biotech, China). The amplified DNA fragments were purified using a PCR Product Purification Kit (Sangon Biotech, Shanghai, China) and sequenced with an ABI 3730XL DNA analyzer at Sangon Biotech Co. Ltd. (Shanghai, China).

The nucleotide and protein sequences were compared with the National Center for Biotechnology Information (NCBI) nucleotide/protein database by BLASTN and BLASTP, respectively. The signal peptide sequence was analyzed by SignalP version 4.0 [31]. Multiple sequence alignments were performed with ClustalW version 2.1. A phylogenetic tree was constructed using the neighbor-joining (NJ) method in MEGA version 5.0 [32]. Confidence in the tree topology was estimated using bootstrap values based on 1000 replicates.

### 4.3. Cloning of AfOTase Gene

The *Af*OTase gene was amplified from *A. faecalis* DSM 16503 genomic DNA (gDNA) by PCR using LA Taq (TaKaRa, Kusatsu, Japan). Two primers, OD-F (CGCGGATCCCAAGCCAGTAATCCCATGATGG, with a BamHI site) and OD-R (CCCAAGCTTCTACGGTTTTTTGTGATCCATC, with a HindIII site) were used for PCR amplification and partial sequencing of the *Af*OTase gene. The gene was ligated into pET-28a (+) which was linearized by BamHI-HF and HindIII-HF (NEB). The vector pET-28a (+) with His_6_-tags at the N-terminus was used for recombinant expression in this study.

### 4.4. Expression of AfOTase in E.coli

The recombinant plasmid was transformed into *E. coli* BL21 (DE3). The recombinant *E. coli* BL21 (DE3) was first inoculated into 3 mL LB medium with 50 μg/mL kanamycin and cultured overnight at 37 °C with shaking at 220 rpm. Then 1 mL of the overnight culture was transferred into 100 mL LB medium with 50 μg/mL kanamycin and grown at 37 °C with shaking at 220 rpm until OD_600_ reached 0.6, then the temperature was decreased to 20 °C and IPTG was added for a final concentration of 0.5 mM. After 16 h, the cells were harvested by centrifugation (4 °C, 4000 × g, 15 min) and stored at −80 °C before purification.

### 4.5. Purification of Recombinant AfOTase

For every gram of induced cells pellet, 5 mL of buffer A (20 mM sodium phosphate, 0.5 M NaCl, pH 7.5), 2 μL of 250 U benzonase (BioLong, Shanghai, China), and 5 μL of 1M MgCl_2_ were added. The cell solution was fully dissolved by stirring and lysed (30 kpsi, 4 °C) by a high-pressure cell disrupter (Constant Systems, Northants, UK) twice, followed by centrifugation (15,000 × g, 4 °C) for 20 min to remove cell debris.

r*Af*OTase with His_6_-tags was purified by Ni^2+^-NTA resin using a biomolecular liquid chromatography system (ÄKTA purifier, GE Healthcare, Boston, MA, USA). The target proteins were eluted with buffer B (20 mM sodium phosphate, 0.5 M NaCl, 250 mM imidazole, pH 7.5). Proteins in the supernatant were precipitated by adding solid ammonium sulfate to 20% and 80% saturation. The fractions precipitating at 20% and 80% saturation were redissolved in 1 mL buffer A and desalted on a Sephadex G-25F column (5mL; GE Healthcare, Boston, MA, USA) with buffer C (20 mM sodium phosphate, 0.1 M NaCl, pH 7.5). The fractions with OTA degrading activity were collected and protein purity was checked by SDS-PAGE.

### 4.6. rAfOTase Activity Assays

r*Af*OTase activity assays followed the method of Dobritzsch et al. [29] with minor variations. The r*Af*OTase assay reaction system consisted of 5 μL sample, 240 μL phosphate buffer (pH 7.5), and 5 μL OTA stock solution (50 μg/mL). Reactions were performed for 10 to 120 min at 37 °C and stopped by adding 250 μL acetonitrile. Then, the sample was centrifuged at 12,000 × g at 4 °C for 10 min. The supernatants were filtered using 0.22 μm syringe filters (Millipore, Cork, Ireland). The concentration of OTA was determined by HPLC equipped with a fluorescence detector (FLD) and the quantitative analysis method was described in detail by Zhang et al. [20].

### 4.7. Biochemical Characterization of rAfOTase

The optimum pH of r*Af*OTase was determined by incubating the recombinant enzyme in a series of 20 mM buffers for 30 min. The pH range from 4.0 to 9.5 was used (20 mM citric acid–phosphate buffer, pH 4.0–7.0; 20 mM Tris–HCl buffer, pH 7.5–8.5; 20 mM glycine–NaOH buffer, pH 9.0–9.5). Similarly, the r*Af*OTase activity assays were done at different temperatures (30–90 °C) to determine the optimum temperature at pH 6.5 for 60 min. When studying the thermal stability of the enzyme, the remaining r*Af*OTase activity was measured after incubating the recombinant enzyme at a certain temperature for 30–150 min. Reactions were stopped as described above. The untreated enzyme was used as the control.

## Supplementary Materials

The following are available online at https://www.mdpi.com/2072-6651/11/9/518/s1, Figure S1. The extract ion chromatogram and MS/MS spectrum of (a1 and b1) ochratoxin A, (a2 and b2) ochratoxin α or (a3 and b3) β-phenylalanine, which the sample processed by r*Af*OTase; Figure S2. Multiple alignment of partial known ochratoxin A degrading enzymes; Figure S3. Plasmid map for *Alcaligenes faecalis* OTase expression vector pET-28a(+)-r*Af*OTase; Figure S4. SDS–PAGE analysis of r*Af*OTase; M, protein size markers; L1, r*Af*OTase was purified by Ni^2+^-NTA resin.
