# Supplementary material for: Heterologous Expression and Characterization of A Novel Ochratoxin A Degrading Enzyme, N-acyl-L-amino Acid Amidohydrolase, from Alcaligenes faecalis
Source: Toxins (Basel). 2019 Sep 6;11(9):518. doi: 10.3390/toxins11090518 (PMC6784128; doi:10.3390/toxins11090518)
Supplement: Supplementary file 1 [file toxins-11-00518-s001.pdf]

toxins

Supplementary Materials: Heterologous Expression and Characterization of A Novel Ochratoxin A Degrading Enzyme, N-acyl-L-amino Acid Amidohydrolase, from *Alcaligenes faecalis*

Honghai Zhang, Yunpeng Zhang, Tie Yin, Jing Wang and Xiaolin Zhang

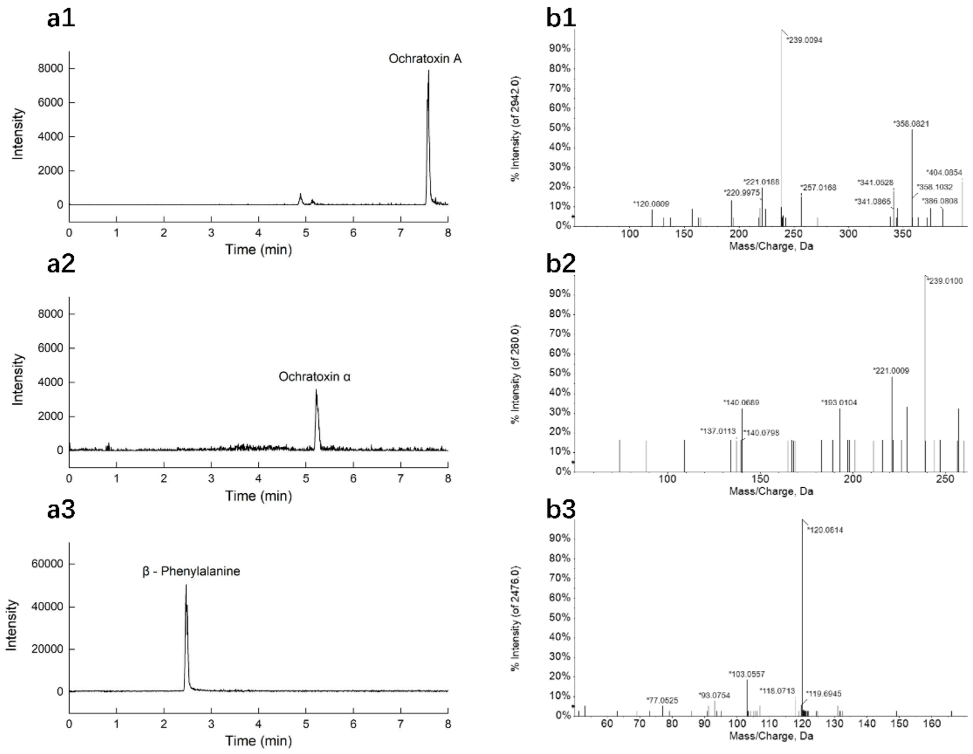

Figure S1. The extract ion chromatogram and MS/MS spectrum of (**a1** and **b1**) ochratoxin A, (**a2** and **b2**) ochratoxin α or (**a3** and **b3**) β-phenylalanine, which the sample processed by r*Af*OTase.

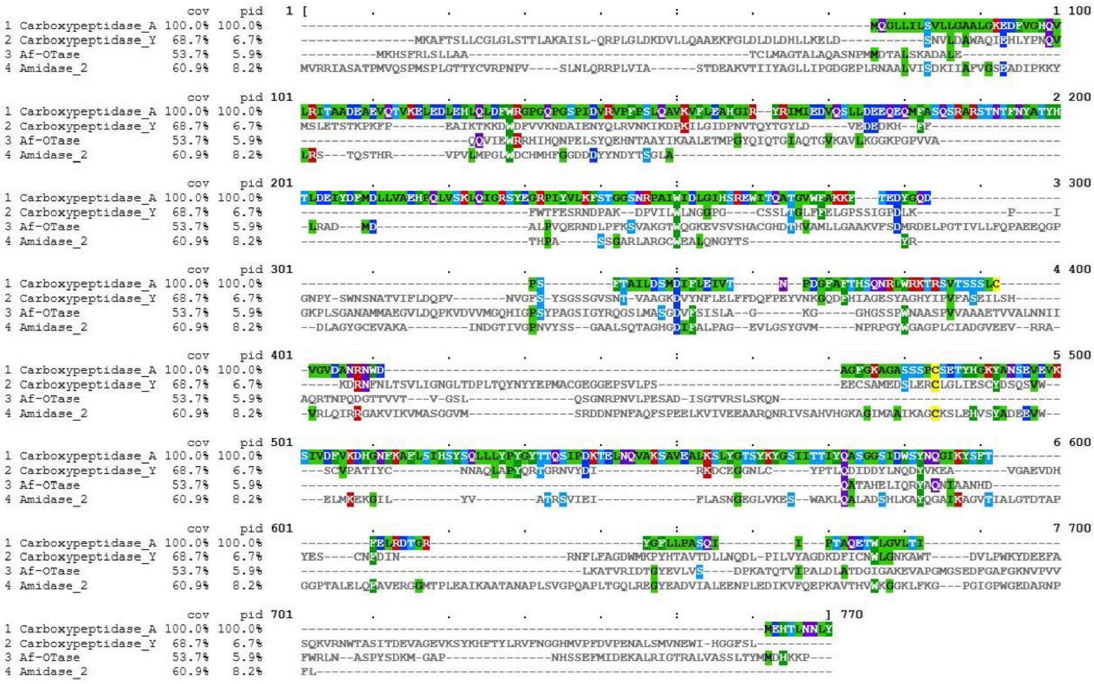

Figure S2. Multiple alignment of partial known ochratoxin A degrading enzymes. 1: Carboxypeptidase A from bovine pancreas; 2: Carboxypeptidase Y from *Saccharomyces cerevisiae*; 3: *Af*OTase from *Alcaligenes faecalis*; 4: Amidase 2 from *Aspergillus niger*.

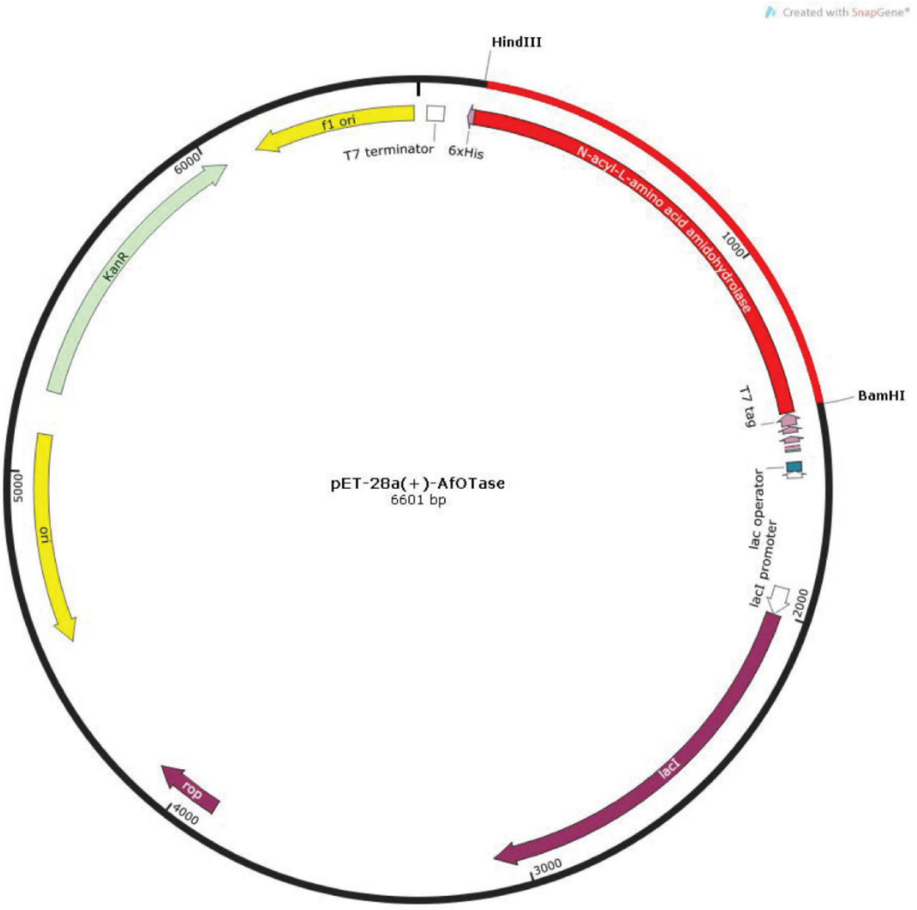

Figure S3. Plasmid map for *Alcaligenes faecalis* OTase expression vector pET-28a(+)-r*Af*OTase.

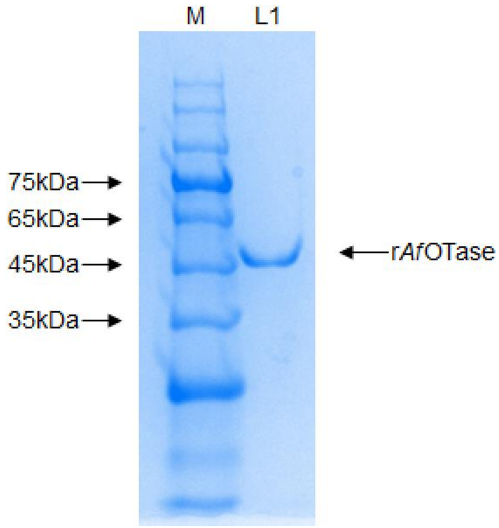

Figure S4. SDS–PAGE analysis of r*Af*OTase; M, protein size markers; L1, r*Af*OTase was purified by Ni^{2+}-NTA resin.

© 2019 by the author. Licensee MDPI, Basel, Switzerland. This article is an open access article distributed under the terms and conditions of the Creative Commons Attribution (CC BY) license (http://creativecommons.org/licenses/by/4.0/).
